# Gesundheitsschutz und Klimawandel erfordern ambitionierte Grenzwerte für Luftschadstoffe in Europa: Stellungnahme zur Revision der Richtlinie über Luftqualität und saubere Luft für Europa der Kommission Environmental Public Health des Robert Koch-Instituts und des Umweltbundesamtes

**DOI:** 10.1007/s00103-023-03755-8

**Published:** 2023-08-21

**Authors:** Annette Peters, Caroline Herr, Gabriele Bolte, Astrid Heutelbeck, Claudia Hornberg, Thomas Kraus, Tobia Lakes, Andreas Matzarakis, Dennis Novak, Doreen Reifegerste, Claudia Traidl-Hoffmann, Hajo Zeeb, Alexandra Schneider, Barbara Hoffmann

**Affiliations:** 1 Institut für Epidemiologie, Helmholtz Zentrum München – Deutsches Forschungszentrum für Gesundheit und Umwelt (GmbH), Ingolstädter Landstr. 1, 85764 Neuherberg, Deutschland; 2 Lehrstuhl für Epidemiologie, Institut für Medizinische Informationsverarbeitung, Biometrie und Epidemiologie, Medizinische Fakultät, Ludwig-Maximilians-Universität München, München, Deutschland; 3 Bayerisches Landesamt für Gesundheit und Lebensmittelsicherheit, München, Deutschland; 4 Institut für Public Health und Pflegeforschung, Abteilung Sozialepidemiologie, Universität Bremen, Bremen, Deutschland; 5 Institut für Arbeits‑, Sozial- und Umweltmedizin, Universitätsklinikum Jena, Jena, Deutschland; 6 Medizinische Fakultät OWL, Universität Bielefeld, Bielefeld, Deutschland; 7 Institut für Arbeits‑, Sozial- und Umweltmedizin, Uniklinik RWTH Aachen, Aachen, Deutschland; 8 Geographisches Institut, Angewandte Geoinformationsverarbeitung, Humboldt-Universität zu Berlin, Berlin, Deutschland; 9 Zentrum für Medizin-Meteorologische Forschung, Deutscher Wetterdienst, Freiburg, Deutschland; 10 Instituts- und Poliklinik für Arbeits‑, Sozial- und Umweltmedizin, Ludwig-Maximilians-Universität Klinikum, München, Deutschland; 11 Fakultät für Gesundheitswissenschaften, Universität Bielefeld, Bielefeld, Deutschland; 12 Medizinische Fakultät – Lehrstuhl für Umweltmedizin, Universitätsklinikum Augsburg, Augsburg, Deutschland; 13 Leibniz-Institut für Präventionsforschung und Epidemiologie – BIPS und Universität Bremen, Bremen, Deutschland; 14 Institut für Arbeits‑, Sozial- und Umweltmedizin, Universitätsklinikum Düsseldorf, Düsseldorf, Deutschland

**Keywords:** Luftschadstoffe, Luftqualität, Gesundheitsschutz, Klimawandel, Krankheitslast, Air pollutants, Air quality, Health protection, Climate change, Burden of disease

## Abstract

Die Weltgesundheitsorganisation (WHO) hat aufgrund wissenschaftlicher Ergebnisse die Richtwerte zur Luftqualität 2021 verschärft. Es wurde eine deutliche Absenkung der Jahresmittelwerte von Feinstaub (Partikelgröße 2,5 µm oder kleiner, PM_2,5_) sowie der Langzeitbelastung gegenüber Stickstoffdioxid (NO_2_) und Ozon (O_3_) empfohlen. Das Mortalitätsrisiko, wie Studien für den Bereich der niedrigen Luftschadstoffkonzentrationen belegen, steigt bereits oberhalb der WHO-Richtwerte an. In Deutschland wurden die WHO-Richtwerte von 2021 für PM_2,5_ und NO_2_ im Jahr 2022 deutlich überschritten.

In diesem Positionspapier geben wir folgende Empfehlungen zur Europäischen Luftqualitätsrichtlinie: (1) Festlegung von verbindlichen Grenzwerten entsprechend WHO 2021, (2) Geltungsbereich der Grenzwerte in ganz Europa, (3) Fortführung und Ausbau der etablierten Ländermessnetze, (4) Ausbau der Luftqualitätsmessungen für ultrafeine Partikel und Rußpartikel, (5) Verknüpfung von Maßnahmen zur Luftreinhaltung und zum Klimaschutz.

Eine Verschärfung der Grenzwerte für Luftschadstoffe erfordert gesellschaftliche und politische Veränderungen unter anderem in den Bereichen Mobilität, Energienutzung und -erzeugung, Stadt- und Raumplanung. Die Umsetzung der WHO-2021-Richtwerte hätte einen volkswirtschaftlichen Nettonutzen von 38 Mrd. € im Jahr.

Ambitionierte Grenzwerte für Luftschadstoffe helfen auch, den Klimawandel und seine gesundheitlichen Auswirkungen einzudämmen. Die Kommission Environmental Public Health hält daher ambitioniertere Grenzwerte für notwendig, um einen effektiven Gesundheitsschutz in Deutschland zu ermöglichen, und fordert, dass Luftschadstoffgrenzwerte entsprechend den WHO-Empfehlungen von 2021 in Europa verbindlich werden.

## Abstract (en)

Based on scientific findings, the World Health Organization (WHO) has recommended stricter guideline values for air quality in 2021. Significant reductions in the annual mean values of particulate matter (particle size 2.5 µm or smaller, PM_2.5_) and long-term exposure to nitrogen dioxide (NO_2_) and ozone (O_3_) were put forward. The risk of mortality already increases above the WHO guideline values, as shown in studies investigating low concentrations of air pollutants. In Germany, the 2021 WHO guideline values for PM_2.5_ and NO_2_ were clearly exceeded in 2022.

In this position paper we give the following recommendations for the European Air Quality Directive: (1) set binding limit values according to WHO 2021, (2) apply the limit values to the whole of Europe, (3) continue and expand the established country-based monitoring networks, (4) expand air quality measurements for ultrafine particles and soot particles, and (5) link air pollution control and climate protection measures.

Stricter limits for air pollutants require societal and political changes in areas such as mobility, energy use and generation, and urban and spatial planning. Implementation according to WHO 2021 would lead to a net economic benefit of 38 billion euros per year.

Ambitious limit values for air pollutants also have an impact on climate change mitigation and its health impacts. The Environmental Public Health commission concludes that more ambitious limit values are crucial to enable effective health protection in Germany and calls for air pollutant limit values in line with the 2021 WHO recommendations to become binding in Europe.

Saubere Luft ist eine wesentliche Voraussetzung für die Gesundheit der Bevölkerung und des Einzelnen. Die Weltgesundheitsorganisation (WHO) hat aufgrund der eindeutigen Ergebnisse von Studien die Richtwerte zur Luftqualität im Jahr 2021 deutlich verschärft ([1]; Tab. 1). Insbesondere wurde eine deutliche Absenkung des Jahresmittelwerts von Feinstaub mit einem aerodynamischen Durchmesser bis 2,5 µm (Particulate Matter: PM_2,5_) auf Werte unterhalb von 5 µg/m^3^ empfohlen. Die WHO empfiehlt zudem eine Absenkung der Langzeitbelastung gegenüber dem gasförmigen Luftschadstoff Stickstoffdioxid (NO_2_) auf unter 10 µg/m^3^ im Jahresmittel und von Ozon (O_3_) auf weniger als 60 µg/m^3^ im max. 8 h-Mittelwert der Hochsaison (6 Monate). Diese Luftschadstoffe treten aufgrund gemeinsamer Quellen sowie chemischer und physikalischer Prozesse zusammen in einem Gemisch auf [2].

**Table 1:** 

		WHO 2005 Luftschadstoff-Richtwerte	WHO 2021 Luftschadstoff-Richtwerte	EU 2008 EU-Standards für Luftschadstoffe	Entwurf EU 2022 Standards für Luftschadstoffe
*PM*_*2,5*_	Jahresmittelwert	10 µg/m^3^	5 µg/m^3^	25 µg/m^3^	10 µg/m^3^
*PM*_*2,5*_	Tagesmittelwert (24 h)	25 µg/m^3^ ^a^	15 µg/m^3^ ^a^	–	25 µg/m^3^ ^b^
*PM*_*2,5*_	3‑Jahres-Mittelwert an Hintergrundstationen (pro NUTS1 Region ^c^)	–	–	–	25 % Reduktion alle 10 Jahre
*PM*_*10*_	Jahresmittelwert	20 µg/m^3^	15 µg/m^3^	40 µg/m^3^	20 µg/m^3^
*PM*_*10*_	Tagesmittelwert (24 h)	50 µg/m^3^ ^a^	45 µg/m^3^ ^a^	50 µg/m^3^ ^d^	45 µg/m^3^ ^b^
*NO*_*2*_	Jahresmittelwert	40 µg/m^3^	10 µg/m^3^	40 µg/m^3^	20 µg/m^3^
*NO*_*2*_	Tagesmittelwert (24 h)	–	25 µg/m^3^ ^a^	–	50 µg/m^3^ ^b^
*O*_*3*_	Hochsaison ^e^	–	60 µg/m^3^	–	100 ^f^
*O*_*3*_	8 h Tagesmaximalwert ^e^	100 µg/m^3^	100 µg/m^3^ ^a^	120 ^g^	120 ^g^

*WHO* World Health Organisation

^a^ 99. Perzentile, nicht überschritten mehr als 3‑ bis 4‑mal pro Kalenderjahr

^b^ Nicht überschritten mehr als 18-mal pro Kalenderjahr

^c^ NUTS1-Regionen sind geographische Einheiten, die in Deutschland den Bundesländern entsprechen

^d^ Nicht überschritten mehr als 35-mal pro Kalenderjahr

^e^ Durchschnitt der täglichen maximalen 8‑Stunden-Mittelwert‑O_3_-Konzentration in den 6 aufeinanderfolgenden Monaten mit dem höchsten 6‑monatig laufenden Mittelwert

^f^ Langzeit-Ziel für den 8‑Stunden-Jahresmittelwert, nicht verbindlich

^g^ Zielwert, nicht verbindlich

Luftschadstoffe erhöhen die Krankheitslast von Atemwegs- und Herz-Kreislauf-Erkrankungen, Diabetes und Demenz bei Erwachsenen und führen auch bei Kindern zu Atemwegserkrankungen und Einschränkungen der gesundheitlichen Entwicklung [3, 4]. Oberhalb der WHO-Richtwerte steigt das Mortalitätsrisiko, wie in großangelegten Studien in Europa und Nordamerika für den Bereich der niedrigen Luftschadstoffkonzentrationen belegt wurde [5–7]. Wichtig ist dabei, dass sowohl durch die Spitzenbelastungen an einzelnen Tagen als auch durch die Jahresmittelkonzentrationen gesundheitliche Auswirkungen beobachtet werden. Aus diesem Grund werden sowohl Kurzzeit- als auch Langzeitgrenzwerte zum effektiven Gesundheitsschutz benötigt.

Die Konzentrationen der meisten Luftschadstoffe sind in den letzten Jahrzehnten in Deutschland gesunken, so dass die bisher geltenden Grenzwerte der Europäischen Union (EU) von 2008 (Tab. 1) eingehalten werden [8]. Die WHO-Richtwerte von 2021 wurden im Gegensatz dazu im Jahr 2022 an 100 % der deutschen Messstellen (223 von 223) für den Jahresmittelwert von PM_2,5_ und an 80 % der Messstellen (425 von 533) für den Jahresmittelwert von NO_2_ überschritten.

Die Fachgesellschaften im Bereich der Lungenerkrankungen und der Umweltepidemiologie fordern eine rasche Umsetzung der WHO-2021-Richtwerte in die Europäische Luftqualitätsrichtlinie [9]. Dieser Forderung schließt sich die Kommission „Environmental Public Health“ nachdrücklich aus gesundheitswissenschaftlicher Sicht an.

Die im Gesetzesentwurf der Europäischen Kommission von 2022 vorgeschlagene deutliche Verschärfung der Grenzwerte für PM_2,5_ und NO_2_ [10] ist aber aus Sicht der Kommission „Environmental Public Health“ für einen effektiven Gesundheitsschutz immer noch unzureichend. Zudem fehlt ein verbindlicher Grenzwert für O_3_. Aus Sicht des Gesundheitsschutzes sind bei der Überarbeitung der Gesetzgebung folgende Aspekte wichtig:

1. Festlegung von verbindlichen Grenzwerten entsprechend den Empfehlungen der WHO von 2021, insbesondere für PM_2,5_ und NO_2_ als Jahres- und 24-Stunden-Mittelwerte sowie als 6‑Monats- und 8‑h-Mittelwerte für O_3_ und die Einhaltung dieser Grenzwerte ab dem Jahr 2030;
2. Geltungsbereich der Grenzwerte an allen Orten in Europa, um einen flächendeckenden Gesundheitsschutz in Deutschland und ganz Europa zu ermöglichen;
3. Fortführung und Ausbau der etablierten Ländermessnetze, um Luftqualität an besonders belasteten Orten im urbanen und ländlichen Umfeld zu erfassen. Die gewonnenen Daten können als Grundlage für Luftreinhaltungsmaßnahmen dienen.
4. Ausbau der Luftqualitätsmessungen für die bisher nicht regulierten ultrafeinen Partikel und Rußpartikel, entsprechend den Empfehlungen der WHO 2021;
5. Verknüpfung von Maßnahmen zur Luftreinhaltung und zum Klimaschutz.

Wenn die Grenzwerte für Luftschadstoffe gesenkt werden, sind für deren Einhaltung gesellschaftliche und politische Veränderungen erforderlich, vor allem in Bezug auf Mobilität, Energienutzung und -erzeugung, Stadt- und Raumplanung sowie Industrie (speziell auch Agrarindustrie). In allen Sektoren ist ein europaweites Umdenken notwendig. Eine strenge Regulierung der Luftschadstoffe entsprechend den Empfehlungen der WHO 2021 würde zu einem volkswirtschaftlichen Nettonutzen von 38 Mrd. € im Jahr und zu einem Anstieg des Bruttosozialprodukts von bis zu 0,44 % im Jahr 2030 in Europa führen [11].

Die Verbesserung der Luftqualität hat neben den direkten positiven Auswirkungen auf die Gesundheit auch wichtige indirekte positive Wirkungen in Hinblick auf die Eindämmung des Klimawandels und seiner gesundheitlichen Folgen. Ambitionierte Grenzwerte für Luftschadstoffe unterstützen technologische Pfade zur Kohlendioxidneutralität und zur Nutzung von erneuerbaren Energien. Niedrigere Luftschadstoffbelastungen dämmen die gesundheitlichen Auswirkungen von Hitze ein, wie aktuelle Analysen für Interaktionen zwischen hohen Lufttemperaturen und -schadstoffen in Bezug auf die Mortalität in Deutschland belegen [12].

Daher wird aus Sicht der Kommission ein begleitendes Monitoring der erreichten Minderungen der Luftschadstoffkonzentrationen gemeinsam mit wetterbasierten Klimawandelindikatoren empfohlen. Idealerweise sollten dafür die bereits seit vielen Jahren existierenden Messstationen mit hochauflösenden Aerosolmessungen in einem Netzwerk integriert werden, da sie insbesondere die Veränderungen in den ultrafeinen Partikeln abbilden. Die Daten könnten bei der Anpassung von Luftreinhaltungsmaßnahmen unterstützen. Die Messstationen der Ländermessnetze sollten durch Messungen der Partikelanzahlkonzentration ergänzt werden, um die Belastung durch ultrafeine Partikel abschätzen zu können.

Auf der Seite der Gesundheitsforschung ist das Monitoring mit Hilfe von täglichen und regional aufgelösten Todesfall- und Krankenhauseinweisungsdaten zur Abschätzung der Krankheitslast erforderlich, die aufgrund von kurzzeitig erhöhten Schadstoffkonzentrationen entsteht. Eine Erfassung der Sichtweise der Bevölkerung im Rahmen von regelmäßigen Gesundheitserhebungen ist notwendig, um die gesundheitlichen und gesellschaftlichen Aspekte abzubilden. Zudem führen die NAKO-Gesundheitsstudie (NAKO) und andere Kohortenstudien detaillierte prospektive Untersuchungen der Krankheitsentstehung in Deutschland durch. Sie bieten eine exzellente Ausgangsposition, um die langfristigen, positiven Auswirkungen der Luftqualitätsverbesserung auf die Gesundheit der Bevölkerung und insbesondere vulnerabler Gruppen abzubilden.

Die Vorschläge der EU-Kommission (Tab. 1) bedeuten eine deutliche Verbesserung gegenüber den bestehenden Grenzwerten, sind aber im Lichte des heutigen Wissenstands nicht in der Lage, die Gesundheit der Bevölkerung und des Einzelnen wirksam zu schützen. Die Kommission „Environmental Public Health“ stellt fest, dass deutlich ambitioniertere Grenzwerte notwendig sind, um einen effektiven Gesundheitsschutz in Deutschland und in ganz Europa zu ermöglichen, und fordert daher, dass Luftschadstoffgrenzwerte entsprechend den WHO-Empfehlungen von 2021 in Europa verbindlich werden.

## Mitglieder der Kommission „Environmental Public Health“

Kommissionsmitglieder: Prof. Dr. Gabriele Bolte (Institut für Public Health und Pflegeforschung, Universität Bremen), Prof. Dr. Caroline Herr (Landesamt für Gesundheit und Lebensmittelsicherheit Bayern), Prof. Dr. Astrid Heutelbeck (Institut für Arbeits‑, Sozial- und Umweltmedizin, Universitätsklinikum Jena), Dr. Henk Hilderink (Rijksinstituut voor Volksgezondheid en Milieu, Hilversum), Prof. Dr. Barbara Hoffmann (Institut für Arbeits‑, Sozial- und Umweltmedizin, Universitätsklinikum Düsseldorf), Prof. Dr. Claudia Hornberg (Medizinische Fakultät OWL, Universität Bielefeld), Prof. Dr. Thomas Kraus (Institut für Arbeits‑, Sozial- und Umweltmedizin, Uniklinik RWTH Aachen), Prof. Dr. Tobia Lakes (Geographisches Institut, Humboldt-Universität zu Berlin), Prof. Dr. Andreas Matzarakis (Zentrum für Medizin-Meteorologische Forschung, Deutscher Wetterdienst Bereich Klima und Umwelt, Freiburg), Dr. Odile Mekel (Landeszentrum Gesundheit Nordrhein-Westfalen), Prof. Dr. Annette Peters (Institut für Epidemiologie, Helmholtz Zentrum München), Dr. Martina Ragettli (Schweizerisches Tropen- und Public Health-Institut, Basel – Allschwil), Prof. Dr. Doreen Reifegerste (Fakultät für Gesundheitswissenschaften, Universität Bielefeld), Prof. Dr. Dennis Nowak (Instituts- und Poliklinik für Arbeits‑, Sozial- und Umweltmedizin, Ludwig-Maximilians-Universität Klinikum München), Dr. Alexandra Schneider (Institut für Epidemiologie, Helmholtz Zentrum München), Prof. Dr. Claudia Traidl-Hoffmann (Medizinische Fakultät – Lehrstuhl für Umweltmedizin, Universität Augsburg), Prof. Dr. Hajo Zeeb (Leibniz-Institut für Präventionsforschung und Epidemiologie – BIPS und Universität Bremen)
